# Interfacial Compatibility and Performance Evaluation of Waste Plastic Aggregate in SBS-Modified Asphalt Mixtures Using Liquid Anti-Stripping Agents

**DOI:** 10.3390/polym18131583

**Published:** 2026-06-25

**Authors:** Joohan Eom, Kyungnam Kim, Jaehyun Lee, Tri Ho Minh Le

**Affiliations:** 1 Department of Civil Engineering, Kunsan National University, Kunsan 54150, Jeonbuk, Republic of Korea; joohan.eom@sgs.com; 2 Pavement Research Division, Korea Expressway Corporation Research Institute, Dong-tansunhwan-daero 17-gil, Hwaseong-si 18489, Gyeonggi-do, Republic of Korea; 3 Construction Materials Testing Center, Business Development, Dongtan Laboratory, SGS Korea Co., Ltd., 20 Dongtansandan 10-gil, Dongtan-myeon, Hwaseong-si 18487, Gyeonggi-do, Republic of Korea; jaehyun.lee@sgs.com; 4 Faculty of Civil Engineering, Nguyen Tat Thanh University, 300A Nguyen Tat Thanh Street, Xom Chieu Ward, Ho Chi Minh City 70000, Vietnam; lhmtri@ntt.edu.vn

**Keywords:** waste plastic aggregate, SBS-modified asphalt, anti-stripping agent, binder adhesion, Hamburg wheel tracking, SCB fracture energy

## Abstract

Waste plastic aggregate (WPA) is a promising recycled material for asphalt mixtures, but its polymeric surface can weaken binder adhesion and increase moisture-related damage, even in SBS-modified systems. Therefore, a clear need exists to identify anti-stripping agents that are compatible with WPA, rather than simply increasing WPA content in asphalt mixtures. This study evaluates the interfacial and mixture-scale performance of SBS-modified asphalt mixtures containing two WPA types, namely coarse WPA and fine WPA, treated with three liquid anti-stripping agents: amine-based agent (AS-Am), organosilane coupling-type adhesion promoter (AS-OS), and ester/surfactant-based wetting agent (AS-Es). The novelty of this study lies in selecting the anti-stripping system based on WPA–binder adhesion compatibility and validating it through moisture, rutting, rheological, and fracture performance. Binder bond strength, tensile bond strength, shear bond strength, indirect tensile strength/tensile strength ratio (ITS/TSR), Hamburg wheel tracking (HWT), multiple stress creep recovery (MSCR), and semi-circular bending (SCB) tests were conducted. AS-OS showed the best overall performance. It increased binder bond strength (BBS) by 52.8% for coarse WPA and 61.5% for fine WPA, while the optimum 0.5% dosage improved tensile bond strength by 81.0% and 97.2%, respectively. AS-OS also increased shear strength by 58.8–68.3% and improved TSR to 89.0% and 86.2%. In HWT, C-OS and F-OS reduced final rut depth by 44.0% and 45.8%, respectively. SCB results further showed higher fracture work, especially for F-OS. The findings indicate that proper anti-stripping chemistry is essential for durable WPA–SBS asphalt mixtures.

## 1. Introduction

Global plastic production and plastic waste generation have increased rapidly during the last two decades. According to the OECD, global plastic production increased from 234 million tonnes in 2000 to 460 million tonnes in 2019, while plastic waste increased from 156 million tonnes to 353 million tonnes over the same period [1]. Only about 9% of plastic waste was ultimately recycled, while about 19% was incinerated and nearly 50% was landfilled [1]. These numbers show that conventional plastic waste management remains insufficient and that high-volume recycling routes are still needed.

Road construction is one possible route for consuming large amounts of recycled materials. Asphalt mixtures are especially attractive because they contain a high aggregate fraction and are produced on a large scale [2]. Recycled plastic can be used in asphalt either as a binder modifier or as a partial aggregate substitute [3]. Among these approaches, WPA is interesting because it can reduce the demand for natural aggregate while providing a practical recycling route for waste plastic in pavement layers.

Lee et al. evaluated WPA in asphalt mixtures using different replacement contents and additive materials, including magnesium, fly ash, and steel slag [4]. Their results showed that 5% WPA could be incorporated without major production or performance problems, while mixtures with 7% WPA or higher failed to satisfy water-resistance requirements. Lee and Le [5] further extended this concept by combining WPA with RAP and sewage sludge bio-oil, reporting that 20% WPA with bio-oil improved durability and reduced rutting by about 42%, while also reducing CO_2_ emissions by 18%. Katla et al. [6] also confirmed the practical limitation of plastic aggregate replacement, showing that mixtures with 2.5% and 5.0% artificial plastic aggregate satisfied volumetric and strength requirements, with rut depth reductions of 12% and 24%, respectively. Recent studies also indicate that WPA can improve sustainability and may enhance rutting resistance, but its use is highly dependent on replacement level, particle type, and moisture resistance [7,8,9,10,11]. Therefore, WPA should not be treated only as a recycled aggregate substitute; its interfacial compatibility with asphalt binder must be controlled to avoid stripping and water-damage problems [12,13,14].

SBS-modified asphalt binder is widely used to improve elastic recovery, high-temperature rutting resistance, and deformation resistance of asphalt mixtures [15,16]. The SBS polymer network can improve binder rheology, but it does not automatically solve the adhesion problem between asphalt binder and polymeric WPA [17,18,19,20]. Therefore, the key issue in WPA–SBS asphalt mixtures is not only binder stiffness or recovery, but whether the SBS binder can form a stable and durable film on the WPA surface.

Ameri et al. [21] investigated nano-organosilane anti-stripping additive and hydrated lime in HMA mixtures under freeze–thaw cycles and reported that both additives improved ITS, TSR, resilient modulus ratio, and fracture energy, with stronger effects for siliceous aggregates. Nazirizad et al. [22] compared hydrated lime and a liquid anti-stripping agent and found that both improved moisture resistance, while the liquid additive increased TSR by about 16%. Alam and Aggarwal [23] also showed that liquid anti-stripping agents reduced moisture susceptibility, and a silicon-based additive gave the best result at a low dosage of 0.05%. More recently, Lu et al. [24] reported that anti-stripping agents improved adhesion between PE-wax-modified asphalt and aggregate, with KH-550 increasing adhesion work by about 16% and improving binder performance. These findings confirm that anti-stripping agents can improve moisture resistance through better binder–aggregate adhesion, but their effectiveness depends on additive chemistry, aggregate type, dosage, and production conditions [25,26,27,28]. This supports the need for a compatibility-based selection approach when applying anti-stripping agents to WPA-containing SBS-modified asphalt mixtures.

Previous studies have evaluated WPA asphalt mixtures mainly through mixture-level properties such as optimum asphalt content, rutting resistance, indirect tensile strength, and tensile strength ratio [4,11,29,30]. Other studies on polymer-modified asphalt and pavement interface materials have shown that bond strength, shear resistance, and fracture energy are also important indicators of interface durability [31,32,33,34,35]. At the same time, technical reviews have cautioned that recycled plastic in asphalt should be used with proper engineering evaluation, because recycling without sufficient performance verification may reduce pavement service life [36,37,38].

Therefore, this study evaluates the compatibility of three liquid anti-stripping agents with two WPA types in SBS-modified asphalt mixtures. Coarse WPA and fine WPA were used to represent different polymeric aggregate forms. The anti-stripping agents included an amine-based liquid anti-stripping agent (AS-Am), an organosilane coupling-type adhesion promoter (AS-OS), and an ester/surfactant-based wetting agent (AS-Es). The main purpose was to identify the most compatible anti-stripping system for WPA-containing SBS mixtures.

The novelty of this study is the compatibility-based selection of anti-stripping agents for WPA–SBS asphalt systems. Instead of evaluating WPA content alone, this work first examines binder–WPA adhesion using binder bond strength, tensile bond strength, shear bond strength, and shear energy. The selected systems are then validated through indirect tensile strength, tensile strength ratio, Hamburg wheel tracking, multiple stress creep recovery, and semi-circular bending tests. This framework allows the anti-stripping agent to be evaluated by adhesion, moisture resistance, rutting behavior, creep recovery, and fracture performance. It should be noted that this study focuses on LDPE-rich recycled waste plastic aggregate produced by extrusion and particle-size control. The findings should not be generalized to all types of plastic aggregates, because plastic type, surface chemistry, filler content, particle texture, and production process can strongly affect binder adhesion and anti-stripping efficiency. Therefore, the conclusions of this work are limited to the WPA materials and anti-stripping systems evaluated in this study.

## 2. Materials and Methods

### 2.1. Research Framework and Experimental Design

The experimental program was divided into two stages. The first stage evaluated the interface compatibility between SBS binder, WPA surfaces, and liquid anti-stripping agents using BBS, tensile bond, shear bond, and shear energy tests. The second stage validated the selected systems at mixture scale using ITS/TSR, HWT, MSCR, and SCB tests. This sequence was used to confirm whether improved interface adhesion could be translated into better moisture, rutting, rheological, and cracking performance.

Two WPA types were used: coarse WPA (WPA-C) and fine WPA (WPA-F). Three liquid anti-stripping agents were evaluated: amine-based agent (AS-Am), organosilane coupling-type adhesion promoter (AS-OS), and ester/surfactant-based wetting agent (AS-Es). The SBS mixture without WPA and anti-stripping agent was defined as SBS-C. Untreated WPA mixtures were denoted as C0 and F0, while treated mixtures were denoted as C-Am, C-OS, C-Es, F-Am, F-OS, and F-Es. For dosage screening, the anti-stripping agents were added at 0.3%, 0.5%, and 0.7% by binder weight.

The screening approach was based on the assumption that WPA performance is controlled by binder–WPA interface stability. Therefore, the anti-stripping agents were first compared using interface adhesion tests before full mixture validation. The best-performing systems from the adhesion and shear tests were then evaluated through ITS/TSR, HWT, MSCR, and SCB tests to confirm their overall performance.

### 2.2. Materials

#### 2.2.1. SBS-Modified Asphalt Binder

An SBS-modified asphalt binder was used as the base binder in this study. The binder was supplied and prepared through the Korean partner laboratory, SK-Binder Chemical Co., Ltd. (Gyeonggi-do, Republic of Korea). The binder was selected because SBS modification is commonly used to improve elastic recovery, high-temperature deformation resistance, and rutting performance of asphalt mixtures. The basic physical and rheological properties of the SBS-modified binder are summarized in Table 1. The same binder was used for all control, WPA, and anti-stripping-treated mixtures to ensure that the observed differences were mainly associated with WPA type and anti-stripping agent compatibility.

The same SBS-modified binder was used in all control, untreated WPA, and anti-stripping-treated mixtures to maintain a consistent binder phase throughout the experimental program. This design reduced binder-related variability and allowed the effects of WPA type and anti-stripping agent chemistry to be compared more directly. Therefore, the observed differences in interface adhesion, moisture resistance, rutting resistance, creep–recovery response, and fracture behavior can be more reliably linked to the WPA–binder compatibility and anti-stripping treatment.

#### 2.2.2. Natural Mineral Aggregates and Filler

Crushed granite aggregates were used as the natural mineral aggregates. Coarse aggregates, fine aggregates, and limestone filler were prepared to satisfy the target gradation for dense-graded asphalt mixture. The natural aggregate was used as the reference mineral surface for evaluating binder adhesion and as the primary aggregate skeleton in the asphalt mixtures. The physical properties of the natural aggregates and filler are listed in Table 2.

The Los Angeles abrasion loss values of the 20 mm and 13 mm coarse aggregates were 25.85% and 32.97%, respectively. The 13 mm aggregate showed a higher abrasion loss than the 20 mm aggregate, but the value remained below the maximum allowable limit of 40% adopted for the dense-graded asphalt mixture aggregate in this study based on the suggestion from Korean Standard of MOLIT [46]. Therefore, both coarse aggregate fractions were considered suitable for mixture preparation. The LA abrasion result was used only to confirm the mechanical quality of the natural aggregate skeleton before partial replacement with WPA.

#### 2.2.3. Waste Plastic Aggregates

**Figure 1:**
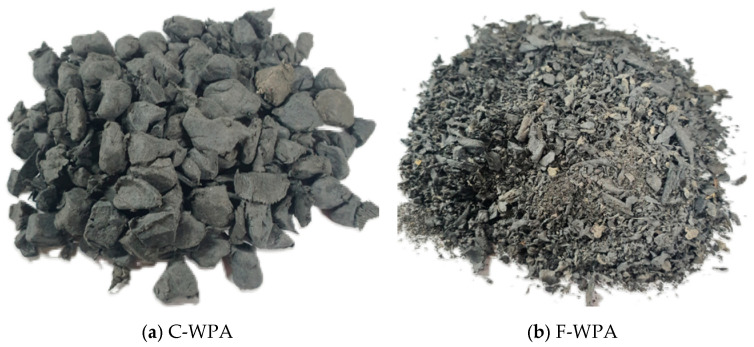
WPA materials used in this study.

The WPA materials were obtained from a recycled plastic aggregate production route developed for asphalt mixture applications (see Figure 1a,b). Waste LDPE-rich plastic was used as the main polymer feedstock, mixed with inorganic additive powder, and processed by high-temperature extrusion molding. The extruded material was then crushed and sieved to produce WPA-C with a nominal size of 5–13 mm and WPA-F with a particle size below 5 mm. This controlled production route was adopted to improve particle shape, size distribution, and handling stability before incorporation into the asphalt mixture [4,5].

During the compounding stage, the dried LDPE-rich plastic flakes were blended with inorganic additive/filler powder before extrusion. The plastic flakes were dried at approximately 80 ± 5 °C to reduce surface moisture before feeding into the extruder. The compounding and extrusion process was carried out using a single-screw extrusion system. The barrel temperature was controlled within 160–180 °C, and the die temperature was maintained at approximately 170 ± 5 °C to ensure stable melting and continuous discharge of the LDPE-rich composite. The screw rotation was kept in a low-to-moderate range of approximately 30–50 rpm to improve mixing uniformity while avoiding excessive thermal degradation of the recycled plastic phase.

The molten LDPE-rich composite was extruded through a controlled die system and discharged as a continuous plastic aggregate precursor. After extrusion, the material was cooled to room temperature to stabilize its shape before crushing. The stabilized extrudate was then mechanically crushed and sieved to obtain two particle-size fractions for asphalt mixture production. The coarse waste plastic aggregate, WPA-C, had a nominal size of 5–13 mm and was used to partially replace the coarse natural aggregate fraction. The fine waste plastic aggregate, WPA-F, had a particle size below 5 mm and was used to partially replace the fine natural aggregate fraction.

This particle-size separation was adopted because WPA-C and WPA-F affect the asphalt mixture structure in different ways. WPA-C mainly influences the coarse aggregate skeleton and load-transfer path, whereas WPA-F has a larger specific surface area and can more strongly affect binder coating, interfacial adhesion, and moisture susceptibility. The final WPA particles had a specific gravity of 1.36, a bulk density of 0.50 g/cm^3^, and low water absorption values of 0.18% for WPA-C and 0.24% for WPA-F. The melting range of the WPA was approximately 108–134 °C, and no visible softening or deformation was observed during asphalt mixing at 160 °C.

Two types of waste plastic aggregate were used: coarse waste plastic aggregate (WPA-C) and fine waste plastic aggregate (WPA-F). WPA-C was used to represent partial replacement of the coarse aggregate fraction, while WPA-F was used to represent partial replacement of the fine aggregate fraction [4,5]. The two WPA types were selected because they can influence the asphalt mixture structure in different ways. WPA-C mainly affects the aggregate skeleton and load transfer, whereas WPA-F has a larger surface area and can strongly affect binder coating and moisture susceptibility.

**Figure 2:**
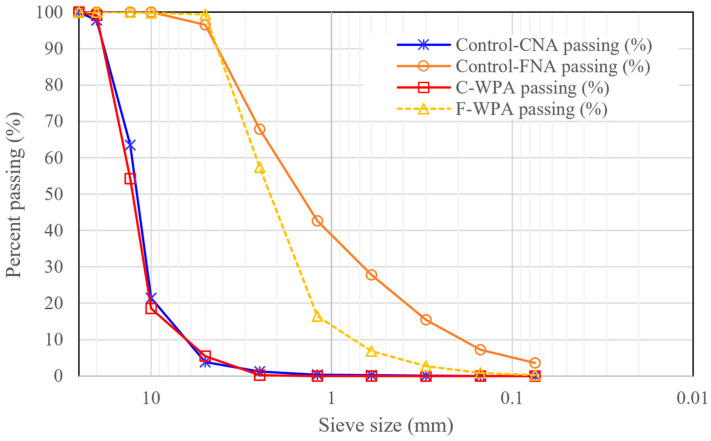
Gradation of aggregates used in this research.

The WPA materials were produced from recycled plastic through extrusion and particle-size control. Additive powder was incorporated during production to improve particle stability and handling. The general properties of the WPA materials are shown in Table 3 and Table 4, while the general gradation of aggregate is presented in Figure 2.

#### 2.2.4. Liquid Anti-Stripping Agents

Three liquid anti-stripping agents were selected to represent different interfacial modification mechanisms as shown in Table 5. The agents were supplied by SK-Binder Chemical Co., Gyeonggi-do, Republic of Korea, as commercial liquid additives for asphalt binder applications. The first agent was an amine-based liquid anti-stripping agent (AS-Am), which represents a conventional adhesion-promoting system [47]. The second agent was an organosilane coupling-type adhesion promoter (AS-OS), selected to improve compatibility between the SBS binder and the polymeric WPA surface [48]. The third agent was an ester/surfactant-based wetting agent (AS-Es), selected to improve coating and binder distribution around WPA particles [49]. The anti-stripping agents were added by binder weight. For dosage screening, 0.3%, 0.5%, and 0.7% were used. Based on the tensile bond strength screening, the optimum dosage was selected for the subsequent shear bond and mixture-level tests.

### 2.3. Anti-Stripping Agent Dosage Design

The liquid anti-stripping agents were incorporated into the SBS-modified binder before aggregate mixing. Three anti-stripping agents were evaluated: AS-Am, AS-OS, and AS-Es. For the dosage screening stage, each agent was added at 0.3%, 0.5%, and 0.7% by binder weight. These dosage levels were selected to identify the optimum dosage and to check whether excessive liquid additive could reduce bonding efficiency.

The optimum dosage of liquid anti-stripping agent should balance adhesion improvement, binder-film stability, and compatibility with the SBS-modified binder. An insufficient dosage may not provide enough polar or surface-active components to improve wetting and adhesion at the WPA–binder interface, leading to limited improvement in moisture resistance. In contrast, excessive dosage may disturb the SBS binder film, reduce interfacial stiffness, or act as a weak boundary layer between the binder and WPA surface. Therefore, the dosage was screened using tensile bond strength before mixture-scale testing. The 0.5% dosage was selected because it produced the highest tensile bond strength for both WPA-C and WPA-F, while the 0.7% dosage showed a slight reduction in bonding efficiency.

The dosage screening was conducted using the tensile bond strength test because this test directly reflects the adhesion capacity between the SBS binder and WPA surface. For each WPA type, the untreated mixture was used as the reference condition. The treated systems were then compared with the untreated WPA mixture to determine the dosage that produced the highest tensile bond strength. Based on the screening results, 0.5% by binder weight was selected as the optimum dosage for the following shear bond, ITS/TSR, HWT, MSCR, and SCB tests.

This dosage design ensured that the final mixture-scale evaluation used the most effective anti-stripping content identified from the interface-level test. It also avoided selecting the anti-stripping agent based only on rutting or moisture performance after full mixture preparation.

The mixing and specimen preparation conditions were controlled to reduce variability caused by temperature, mixing time, and coating conditions as presented in Table 6. WPA was preheated at a lower temperature than natural aggregate to avoid thermal deformation, while the SBS binder and natural aggregates were heated to conventional asphalt mixing temperatures. The liquid anti-stripping agents were pre-blended with the SBS binder before aggregate mixing to ensure uniform dispersion.

The asphalt binder content was fixed at 5.4% for all mixtures. This value was selected from the mix design of the control dense-graded SBS-modified asphalt mixture and was used as the reference binder content in this study. The same binder content was then applied to the untreated WPA mixtures and anti-stripping-treated WPA mixtures to allow a direct comparison of WPA type and anti-stripping agent chemistry. This approach was adopted because the main objective of this study was to evaluate WPA–SBS binder compatibility and the effectiveness of liquid anti-stripping agents, rather than to optimize the binder content of each mixture separately.

The WPA was preheated at a lower temperature than the natural aggregate to avoid thermal deformation as shown in Table 7. The liquid anti-stripping agents were pre-blended with the SBS binder before mixing to ensure uniform dispersion. The same mixing and compaction conditions were applied to all mixtures to isolate the effects of WPA type and anti-stripping agent.

### 2.4. Testing Methods

**Figure 3:**
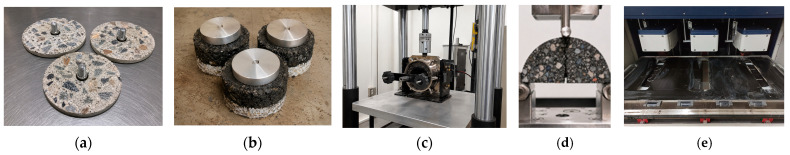
Experimental setup and specimen preparation for interface adhesion and mixture performance evaluation: (**a**) preparation of specimens for BBS test; (**b**) pull-off test; (**c**) shear bond strength test; (**d**) SCB test; and (**e**) HWT test.

The testing program was divided into two stages. Stage 1 focused on interface adhesion between SBS binder and WPA surfaces, including BBS, tensile bond strength, shear bond strength, and shear energy analysis. Stage 2 evaluated mixture-scale performance using ITS/TSR, MSCR, and SCB tests, and HWT test as shown in Figure 3. At least three replicate specimens were prepared for each test condition, and the average value was used for analysis.

#### 2.4.1. Stage 1: Interface Adhesion Evaluation

The BBS test was conducted to evaluate the direct adhesion between SBS binder and aggregate/WPA surfaces. The test followed the principle of AASHTO TP 91 [50]. Natural aggregate, WPA-C, and WPA-F surfaces were cleaned and dried before bonding with the pull-off stub. The SBS binder or SBS binder containing anti-stripping agent was applied between the surface and the metal stub. After conditioning at 25 °C for 24 h, the specimen was pulled at a constant loading rate until failure. The BBS value was calculated using Equation (1):

(1) $$BBS=\frac{P_{max}}{A}$$

where BBS is the binder bond strength (kPa), $P_{max}$ is the maximum pull-off load (N), and A is the bonded area (mm^2^).

The tensile bond strength test was used to determine the optimum dosage of each liquid anti-stripping agent. The test followed the general pull-off adhesion principle of ASTM D4541 [51]. The anti-stripping agents were added at 0.3%, 0.5%, and 0.7% by binder weight. A circular steel dolly was bonded to the prepared WPA–SBS interface and conditioned before testing. The tensile load was applied vertically until separation occurred. The tensile bond strength was calculated using Equation (2):

(2) $$TBS=\frac{P_{max}}{A}$$

where TBS is the tensile bond strength (MPa), $P_{max}$ is the peak tensile load (N), and A is the bonded area (mm^2^). The failure mode was visually classified as adhesive, cohesive, or mixed failure.

The shear bond strength test was conducted to evaluate the resistance of the WPA–SBS interface under shear loading based on AASHTO TP114 [52]. The test followed the concept of direct shear bond evaluation commonly used for pavement interface studies. The bonded interface specimen was placed in a shear fixture and loaded at a constant displacement rate of 50 mm/min at 25 °C. The peak shear load and displacement at peak load were recorded. The shear bond strength was calculated using Equation (3):

(3) $$SBS_{h}=\frac{P_{s}}{A}$$

where $SBS_{h}$ is the shear bond strength (MPa), $P_{s}$ is the peak shear load (N), and A is the shear interface area (mm^2^).

The shear energy was calculated from the area under the shear load–displacement curve up to failure. This parameter was used to evaluate the resistance of the interface against progressive shear damage, not only peak strength. The shear energy was calculated using Equation (4):

(4) $$E_{s}=\int_{0}^{\delta_{f}}P(\delta )\;d\delta$$

where $E_{s}$ is the shear energy (N·mm), P(δ) is the shear load at displacement δ, and $\delta_{f}$ is the displacement at failure. The shear rise factor was also determined from the initial slope up to peak load to compare the initial stiffness of the treated interfaces.

#### 2.4.2. Stage 2: Mixture-Scale Performance Evaluation

The ITS test was conducted to evaluate tensile strength and moisture susceptibility of compacted asphalt mixtures based on AASHTO T 283 [53]. Cylindrical specimens with a diameter of 100 mm and height of approximately 63.5 mm were prepared. Dry specimens were tested at 25 °C, while conditioned specimens were subjected to moisture conditioning before testing. The loading rate was 50 mm/min. The ITS was calculated using Equation (5):

(5) $$ITS=\frac{2P}{\pi Dt}$$

where ITS is the indirect tensile strength (MPa), P is the peak load (N), D is the specimen diameter (mm), and t is the specimen thickness (mm). The tensile strength ratio (TSR) was calculated using Equation (6):

(6) $$TSR=\frac{ITS_{conditioned}}{ITS_{dry}}\times 100$$

where $ITS_{conditioned}$ and $ITS_{dry}$ are the indirect tensile strengths of conditioned and dry specimens, respectively.

The HWT test was used to evaluate rutting and moisture-related deformation under repeated wheel loading based on AASHTO T 324 [54]. Compacted slab specimens were tested in water at 50 °C. The wheel load was applied repeatedly up to 20,000 cycles, and rut depth was recorded throughout the test. The deformation curve was interpreted in stages: initial densification, stable rutting accumulation, and possible stripping-related acceleration.

The multiple stress creep recovery (MSCR) test was conducted to evaluate the creep–recovery response of the SBS binder systems. The test followed the principle of AASHTO T350 [55] using stress levels of 0.1 and 3.2 kPa. Each cycle consisted of 1 s creep loading and 9 s recovery. The non-recoverable creep compliance and percent recovery were calculated from the strain response. The non-recoverable creep compliance was calculated using Equation (7):

(7) $$J_{nr}=\frac{\varepsilon_{nr}}{\sigma }$$

where $J_{nr}$ is the non-recoverable creep compliance (1/kPa), $\varepsilon_{nr}$ is the non-recovered strain after recovery, and σ is the applied stress. The recovery percentage was calculated using Equation (8):

(8) $$R=\frac{\varepsilon_{p}-\varepsilon_{nr}}{\varepsilon_{p}}\times 100$$

where R is the recovery (%), $\varepsilon_{p}$ is the peak strain after creep loading, and $\varepsilon_{nr}$ is the residual strain after recovery.

The semi-circular bending (SCB) test was conducted to evaluate cracking resistance and fracture behavior based on ASTM D8044 [56]. Semi-circular asphalt specimens were cut from compacted cylindrical specimens and notched at the center of the flat edge. The specimens were loaded at 25 °C using a vertical loading head under displacement control. The peak load, displacement at peak load, and post-peak response were recorded. The work of fracture was calculated as the area under the load–displacement curve using Equation (9):

(9) $$W_{f}=\int_{0}^{\delta_{f}}P(\delta )\;d\delta$$

where $W_{f}$ is the work of fracture (kN·mm), P(δ) is the applied load at displacement δ, and $\delta_{f}$ is the final displacement.

In this study, the work of fracture was used as a fracture energy index because it reflects both crack initiation and post-peak crack propagation resistance.

To better distinguish the effect of WPA itself from the effect of anti-stripping treatment, the main performance indicators were normalized to a 0–1 scale, as summarized in Table 8. For properties where a higher value indicates better performance, including BBS, tensile bond strength, shear strength, shear energy, TSR, and SCB fracture work, the normalized index was calculated using Equation (10):

(10) $$I_{ij}=\frac{X_{ij}-X_{j,min}}{X_{j,max}-X_{j,min}}$$

For properties where a lower value indicates better performance, including HWT rut depth and MSCR accumulated strain, the normalization was reversed using Equation (11):

(11) $$I_{ij}=\frac{X_{j,max}-X_{ij}}{X_{j,max}-X_{j,min}}$$

where $I_{ij}$ is the normalized index of mixture i for performance indicator j, $X_{ij}$ is the measured value, and $X_{j,min}$ and $X_{j,max}$ are the minimum and maximum values of that indicator among the compared mixtures. Therefore, a normalized value close to 1.0 indicates better performance, while a value close to 0 indicates weaker performance. The average performance index of each mixture was calculated using Equation (12):

(12) $$I_{avg,i}=\frac{1}{m_{i}}\sum_{j=1}^{m_{i}}I_{ij}$$

where $I_{avg,i}$ is the average normalized index of mixture i, and $m_{i}$ is the number of available performance indicators used for that mixture.

**Figure 4:**
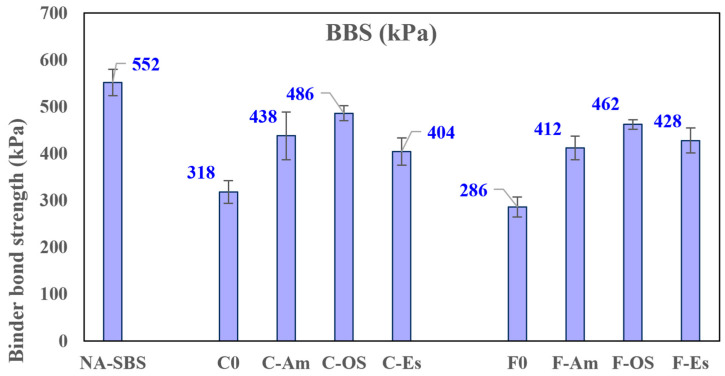
Binder bond strength of SBS binder on natural aggregate and WPA surfaces with different liquid anti-stripping agents.

**Figure 5:**
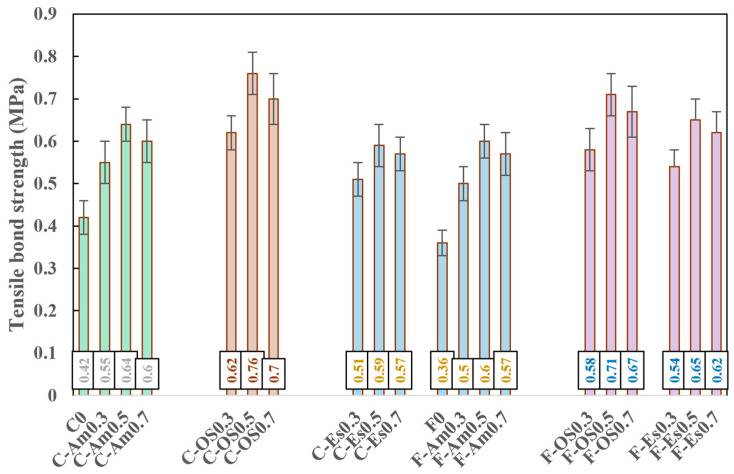
Tensile bond strength of WPA–SBS binder systems with different liquid anti-stripping agents and dosages.

**Figure 6:**
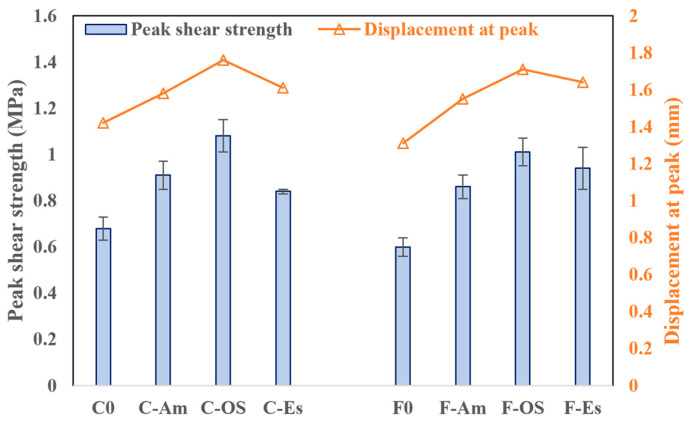
Peak shear strength and displacement at peak load of WPA–SBS binder systems with different liquid anti-stripping agents.

**Figure 7:**
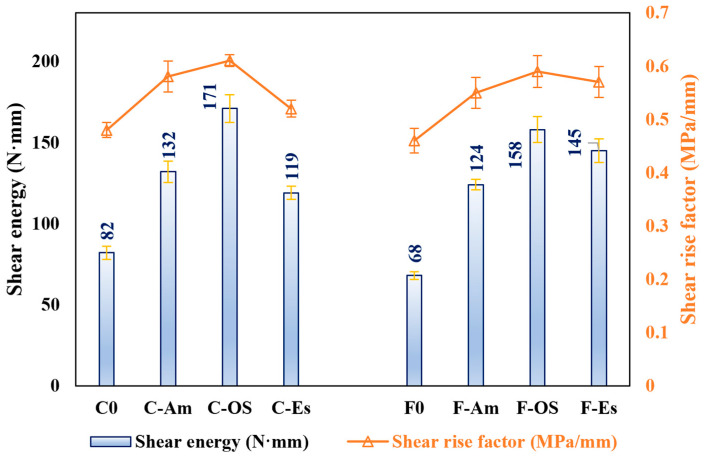
Shear energy and shear rise factor of WPA–SBS binder systems with different liquid anti-stripping agents.

It should be noted that at least three replicate specimens were prepared for each test condition. For scalar performance indicators, including BBS, tensile bond strength, shear bond strength, shear energy, shear rise factor, ITS, TSR, rut depth, and SCB fracture parameters, the results are reported as mean values. Error bars in Figure 4, Figure 5, Figure 6 and Figure 7 represent the standard deviation of replicate measurements and were used to indicate the variability among test specimens. For response-curve results, including MSCR creep–recovery curves, HWT deformation curves, and SCB load–displacement curves, point-by-point error bars were not plotted to maintain figure readability. These curve-based tests were interpreted using the overall response trend and derived performance parameters.

## 3. Results and Discussion

### 3.1. Binder Bond Strength of SBS Binder on WPA Surfaces

Figure 4 compares the binder bond strength (BBS) of the SBS binder on natural aggregate and WPA surfaces. The NA-SBS specimen showed the highest BBS value of 552 kPa. In contrast, the untreated WPA systems showed much lower bond strength, with 318 kPa for C0 and 286 kPa for F0. These values represent reductions of approximately 42.4% and 48.2%, respectively, compared with NA-SBS. This confirms that the WPA surface weakened the binder–aggregate interface, especially in the fine WPA system.

The lower BBS of F0 suggests that fine WPA is more sensitive to adhesion loss than coarse WPA. This may be related to its larger surface area and greater demand for stable binder-film formation. The polymeric nature of WPA may also reduce surface affinity with the SBS binder compared with conventional mineral aggregate.

The addition of liquid anti-stripping agents improved the BBS of both WPA systems. For coarse WPA, AS-Am, AS-OS, and AS-Es increased the BBS by 37.7%, 52.8%, and 27.0%, respectively, compared with C0. For fine WPA, the corresponding improvements were 44.1%, 61.5%, and 49.7% compared with F0. This indicates that all three agents enhanced the binder–WPA interface, although their effectiveness differed.

Among the three agents, AS-OS produced the highest BBS values for both WPA-C and WPA-F, reaching 486 and 462 kPa, respectively. This suggests that the organosilane coupling-type adhesion promoter provided the most effective compatibility improvement. The improvement is likely associated with better wetting and more stable binder-film formation at the WPA surface [48].

These results are broadly consistent with previous pavement-interface studies, where adhesion-promoting or polymer-modified asphalt systems improved bond strength when surface compatibility was a limiting factor. Still, the treated WPA systems did not fully reach the BBS level of NA-SBS. Therefore, anti-stripping treatment improved the inherent adhesion weakness of WPA, but did not completely remove it. Based on this result, AS-OS was selected as the most promising agent for the following tensile bond, shear bond, and mixture-level validation tests.

### 3.2. Effect of Anti-Stripping Dosage on Tensile Bond Strength

Figure 5 presents the tensile bond strength of the WPA–SBS binder systems with different anti-stripping agent dosages. The untreated reference specimens showed the lowest tensile bond strength, with 0.42 MPa for C0 and 0.36 MPa for F0. This confirms that the fine WPA system had a weaker tensile adhesion response than the coarse WPA system, which is consistent with the BBS results in Section 3.1.

For WPA-C, all anti-stripping agents improved the tensile bond strength compared with C0. The AS-Am system increased from 0.42 MPa to 0.64 MPa at 0.5% dosage, while AS-Es increased to 0.59 MPa. The highest value was obtained for C-OS0.5, reaching 0.76 MPa, corresponding to an improvement of approximately 81.0% compared with C0.

A similar trend was observed for WPA-F. The tensile bond strength increased from 0.36 MPa for F0 to 0.60 MPa for F-Am0.5, 0.71 MPa for F-OS0.5, and 0.65 MPa for F-Es0.5. The improvement of F-OS0.5 was approximately 97.2% compared with F0, indicating that AS-OS was particularly effective for the fine WPA system.

For all three anti-stripping agents, the 0.5% dosage produced the highest tensile bond strength, while the 0.7% dosage caused a slight reduction. This indicates that a moderate dosage improved wetting and binder-film stability, but excessive liquid agent may disturb the SBS binder film or reduce interfacial stiffness [57]. At a moderate dosage, AS-OS can improve wetting and binder-film stability at the WPA–SBS interface. However, excessive liquid additive may form a weak boundary layer, disturb the SBS binder film, or reduce local interfacial stiffness. These effects can lower the effective bond strength even when more anti-stripping agent is added. This trend agrees with related adhesion studies where an optimum application amount exists and excessive interfacial material can reduce bond efficiency.

Among the tested systems, AS-OS at 0.5% showed the best tensile adhesion for both WPA-C and WPA-F. Therefore, 0.5% was selected as the optimum anti-stripping dosage for the subsequent shear bond and mixture-level performance evaluations.

### 3.3. Shear Bond Strength and Deformation Tolerance of WPA–SBS Binder Interface

Figure 6 compares the peak shear strength and displacement at peak load of the WPA–SBS binder systems. The untreated WPA specimens showed the lowest shear resistance, with 0.68 MPa for C0 and 0.60 MPa for F0. These results indicate that the WPA–SBS interface was vulnerable under shear loading when no anti-stripping agent was used.

The addition of anti-stripping agents improved the shear bond strength for both WPA types. For WPA-C, the peak shear strength increased from 0.68 MPa to 0.91 MPa with AS-Am, 1.08 MPa with AS-OS, and 0.84 MPa with AS-Es. These correspond to improvements of 33.8%, 58.8%, and 23.5%, respectively, compared with C0. For WPA-F, the improvement was more pronounced. The peak shear strength increased from 0.60 MPa to 0.86 MPa, 1.01 MPa, and 0.94 MPa for AS-Am, AS-OS, and AS-Es, respectively.

The displacement at peak also increased after anti-stripping treatment. For WPA-C, C-OS showed the highest displacement at peak, increasing from 1.42 mm for C0 to 1.76 mm. For WPA-F, F-OS reached 1.71 mm, compared with 1.31 mm for F0. This indicates that the treated interfaces could tolerate larger deformation before shear failure, not only higher peak load.

Among the three agents, AS-OS produced the best shear performance for both WPA-C and WPA-F. The failure mode also changed from mainly adhesive failure in C0 and F0 to mixed or cohesive/mixed failure after treatment. This trend agrees with the BBS and tensile bond results, confirming that AS-OS provided the most effective improvement in WPA–SBS interfacial compatibility. The improvement is likely associated with better binder film stability and stronger resistance to shear-induced debonding at the polymeric WPA surface [4].

The change in failure mode provides additional evidence of improved WPA–SBS interfacial compatibility. In the untreated WPA systems, adhesive failure indicates that separation mainly occurred at the WPA–binder interface, reflecting weak interfacial bonding. After anti-stripping treatment, the failure mode shifted toward mixed or cohesive/mixed failure. This means that the treated interface was no longer the weakest region, and part of the damage occurred within the binder film or bonded zone. Therefore, the failure-mode transition supports the improvement in bond strength and shear resistance after anti-stripping treatment.

In addition to peak shear strength, the shear energy and shear rise factor provide further evidence of improved interfacial resistance shown in Figure 7. The untreated mixtures showed the lowest shear energy, with 82 N·mm for C0 and 68 N·mm for F0. After anti-stripping treatment, the shear energy increased markedly for all systems. C-OS reached the highest value of 171 N·mm, corresponding to an increase of 108.5% compared with C0, while F-OS reached 158 N·mm, corresponding to an increase of 132.4% compared with F0. The higher shear rise factor of the treated mixtures also indicates a stiffer and more stable initial interface before peak loading. These results show that AS-OS not only increased the maximum shear resistance, but also improved the energy absorption capacity of the WPA–SBS interface before failure. This supports the selection of AS-OS as the most effective adhesion-promoting agent for the following mixture-level validation tests.

The better performance of AS-OS can be related to its dual interfacial function. The silane-related groups may interact with polar sites or inorganic filler components on the WPA surface, while the organic functional segment can improve compatibility with the SBS-modified binder. Its low viscosity may also help wet the WPA surface and form a more continuous binder film. As a result, AS-OS reduced adhesive debonding and improved BBS, tensile bond strength, shear bond strength, and shear energy more effectively than AS-Am and AS-Es. This mechanism is proposed from the mechanical results, and future surface analyses such as contact angle, FTIR, SEM, or surface free energy tests are still needed for direct confirmation.

### 3.4. MSCR Creep–Recovery Response of Selected WPA–SBS Binder Systems

**Figure 8:**
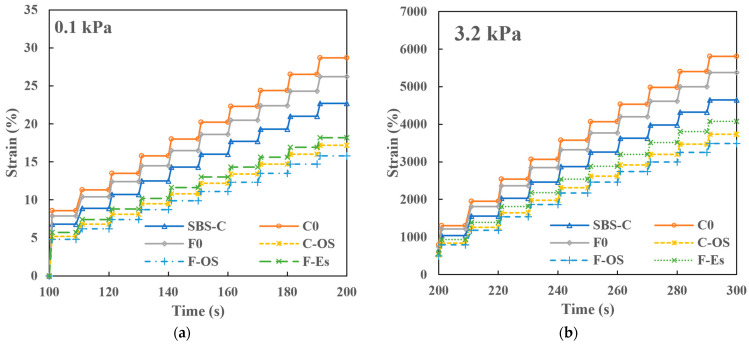
MSCR creep–recovery strain response of selected WPA–SBS binder systems at (**a**) 0.1 and (**b**) 3.2 kPa.

Figure 8 shows the MSCR creep–recovery strain response of the selected WPA–SBS binder systems under 0.1 and 3.2 kPa stress levels. At 0.1 kPa, the untreated WPA systems produced higher accumulated strain than the SBS control. The final strain increased from 22.7 for SBS-C to 28.7 for C0 and 26.2 for F0, corresponding to increases of approximately 26.4% and 15.4%, respectively. This indicates that the WPA systems without anti-stripping treatment were more susceptible to creep deformation, especially the coarse WPA mixture.

The AS-OS-treated systems showed a clear reduction in strain accumulation. At 0.1 kPa, C-OS and F-OS reached final strain values of 17.2 and 15.8, respectively. Compared with the corresponding untreated mixtures, C-OS reduced the final strain by approximately 40.1%, while F-OS reduced it by approximately 39.7%. F-Es also improved the response, reaching a final strain of 18.2, but its performance remained slightly lower than F-OS. This confirms that the OS-type adhesion promoter was more effective in preserving the creep–recovery behavior of the SBS-modified system.

At the higher stress level of 3.2 kPa, the same trend was more evident. C0 and F0 showed the largest strain accumulation, reaching 5810 and 5380 at 300 s. In contrast, C-OS and F-OS reduced the final strain to 3730 and 3490, corresponding to reductions of approximately 35.8% and 35.1%, respectively. The F-Es mixture showed an intermediate response, with a final strain of 4080. This suggests that AS-Es provided some improvement, but the OS-treated systems offered better resistance under high stress.

The improved MSCR response of C-OS and F-OS may be related to better interfacial stability between the SBS binder and WPA surface. A more stable binder film can reduce local slippage and help maintain the elastic recovery contribution of the SBS network during repeated creep–recovery loading. This explanation is consistent with the BBS, tensile bond, and shear bond results, where AS-OS also showed the strongest adhesion improvement.

The MSCR results also agree with previous studies on polymer-modified asphalt binders, where improved binder elasticity and lower non-recoverable deformation are generally associated with better rutting resistance [20]. In this study, the untreated WPA systems weakened the creep–recovery response, while AS-OS compensated for this drawback. Therefore, the MSCR results support the use of AS-OS as the most effective anti-stripping agent for maintaining both adhesion compatibility and rheological stability in WPA-containing SBS-modified asphalt mixtures.

The lower accumulated strain of C-OS and F-OS indicates that improved WPA–binder interfacial stability helped limit local slippage and non-recoverable deformation during repeated creep–recovery loading. This behavior is important for rutting resistance because permanent deformation in asphalt mixtures can develop when the binder film and aggregate interface cannot recover after repeated traffic loading. Therefore, the MSCR results support the HWT findings by showing that AS-OS helped preserve the elastic recovery and deformation resistance of the SBS-modified WPA mixtures.

### 3.5. Indirect Tensile Strength and Moisture Susceptibility

**Figure 9:**
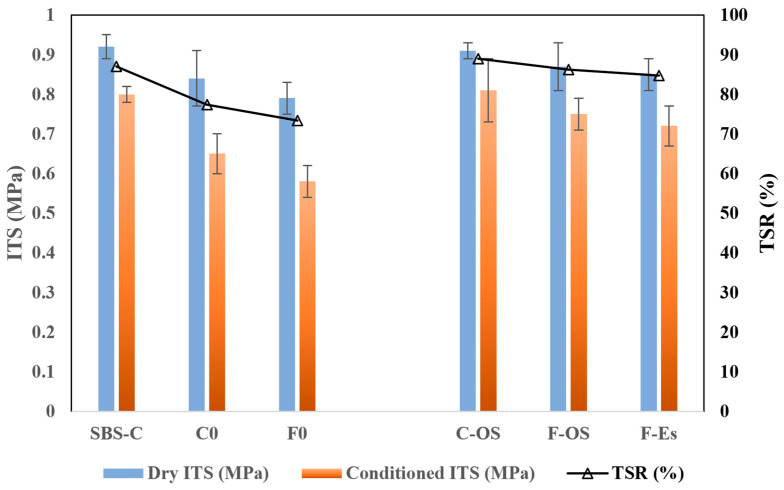
Dry ITS, conditioned ITS, and TSR values of selected WPA–SBS asphalt mixtures.

Figure 9 presents the dry ITS, conditioned ITS, and TSR results of the selected WPA–SBS asphalt mixtures. The SBS-C mixture showed a dry ITS of 0.92 MPa and a conditioned ITS of 0.80 MPa, giving a TSR of 87.0%. This confirms that the SBS control mixture maintained good tensile strength after moisture conditioning, with only 13.0% strength loss. The untreated WPA mixtures showed lower moisture resistance than the SBS control. C0 had a dry ITS of 0.84 MPa and a conditioned ITS of 0.65 MPa, resulting in a TSR of 77.4%. F0 showed the weakest moisture response, with a dry ITS of 0.79 MPa, a conditioned ITS of 0.58 MPa, and a TSR of 73.4%. Compared with SBS-C, the TSR decreased by 9.6 percentage points for C0 and 13.6 percentage points for F0. This indicates that the untreated WPA surface increased moisture susceptibility, especially for the fine WPA system.

The addition of AS-OS improved the moisture resistance of both WPA mixtures. C-OS reached a TSR of 89.0%, which was slightly higher than SBS-C and 11.6 percentage points higher than C0. F-OS increased the TSR from 73.4% to 86.2%, corresponding to a 12.8 percentage-point improvement over F0. These results show that AS-OS effectively reduced moisture-induced strength loss and improved coating retention around the WPA particles. The F-Es mixture also improved the TSR of the fine WPA system, reaching 84.7%. This value was lower than F-OS but still much higher than F0. The result indicates that AS-Es provided moderate improvement in binder coating and moisture resistance, although its effect was not as strong as AS-OS.

The observed trend agrees with the earlier BBS, tensile bond, shear bond, and MSCR results. Improved interfacial adhesion appears to reduce moisture-related debonding and helps the SBS binder maintain a more stable film around the WPA surface. These findings are consistent with related moisture-susceptibility studies, where better binder–aggregate affinity generally leads to higher retained tensile strength. Based on the ITS and TSR results, AS-OS remained the most effective anti-stripping system for both coarse and fine WPA mixtures.

### 3.6. Hamburg Wheel Tracking Performance

**Figure 10:**
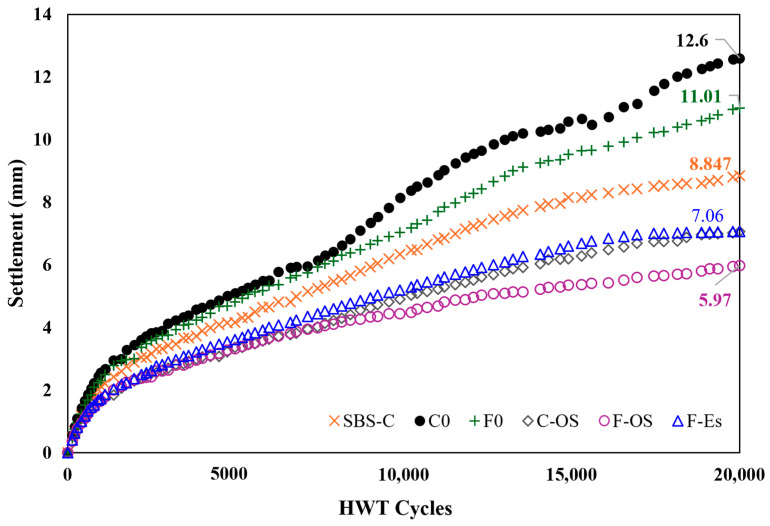
Hamburg wheel tracking deformation curves of selected WPA–SBS asphalt mixtures.

Figure 10 presents the HWT deformation curves of the selected WPA–SBS asphalt mixtures. The deformation response can be divided into three stages: the initial seating stage up to about 1000 cycles, the stable rutting accumulation stage from about 1000 to 10,000 cycles, and the later moisture-damage stage after approximately 10,000–11,000 cycles. This stage-based interpretation is useful because HWT performance is controlled not only by final rut depth, but also by how quickly deformation develops during loading.

During the first 1000 cycles, all mixtures showed rapid deformation. This early deformation is mainly related to specimen seating, aggregate rearrangement, and initial densification under the wheel load. At 985 cycles, SBS-C reached 2.12 mm, while C0 and F0 reached 2.56 and 2.31 mm, respectively. The treated mixtures showed lower early deformation, with 1.64 mm for C-OS, 1.66 mm for F-OS, and 1.73 mm for F-Es. This indicates that the anti-stripping-treated WPA systems had a more stable initial structure, although all mixtures experienced some early compaction.

From approximately 1000 to 5000 cycles, the differences among the mixtures became clearer. At 4980 cycles, C0 reached 5.10 mm and F0 reached 4.82 mm, while SBS-C reached 4.17 mm. In contrast, C-OS, F-OS, and F-Es showed lower deformation values of 3.36, 3.32, and 3.61 mm, respectively. Compared with the untreated WPA systems, the OS-treated mixtures already reduced rut depth by about 34.2% for coarse WPA and 31.1% for fine WPA at this stage. This suggests that improved adhesion and binder-film stability helped slow down rutting accumulation after the initial seating phase.

At around 10,000 cycles, the untreated WPA mixtures continued to accumulate deformation at a higher rate. At 10,220 cycles, C0 reached 8.37 mm and F0 reached 7.18 mm, compared with 6.47 mm for SBS-C. The treated mixtures remained lower, with 5.03 mm for C-OS, 4.48 mm for F-OS, and 5.30 mm for F-Es. This shows that the benefit of anti-stripping treatment became more evident under repeated water–wheel loading. F-OS showed the lowest deformation at this stage, indicating better resistance to combined rutting and moisture-related damage.

After about 11,000 cycles, C0 showed a more pronounced increase in rut depth, suggesting the onset of stripping-related instability or accelerated internal damage. At 20,000 cycles, C0 reached 12.60 mm, while F0 reached 11.01 mm. These values were 42.4% and 24.4% higher than SBS-C, respectively. The sharper increase in C0 indicates that the untreated coarse WPA system was more vulnerable during the later loading stage, while F0 showed a weaker but more gradual deformation trend.

The AS-OS-treated mixtures showed the best later-stage performance. At 20,000 cycles, C-OS reached 7.06 mm, representing a 44.0% reduction compared with C0. F-OS showed the lowest final rut depth of 5.97 mm, corresponding to a 45.8% reduction compared with F0. F-Es also reduced the final deformation to 7.07 mm, which was 35.8% lower than F0, but still higher than F-OS. These results indicate that AS-OS provided the most effective protection against moisture-rutting damage, while AS-Es gave moderate improvement for fine WPA.

The HWT results are consistent with the earlier adhesion and moisture-resistance results. Mixtures with stronger BBS, tensile bond strength, and shear bond strength generally showed lower rut depth and more stable deformation curves. This suggests that better WPA–SBS binder compatibility reduced binder film loss and delayed moisture-induced weakening during wheel tracking. Therefore, the stage-based HWT analysis confirms that AS-OS was the most effective anti-stripping agent for improving the long-term deformation resistance of WPA-containing SBS-modified asphalt mixtures.

### 3.7. SCB Fracture Behavior of Selected WPA–SBS Asphalt Mixtures

**Figure 11:**
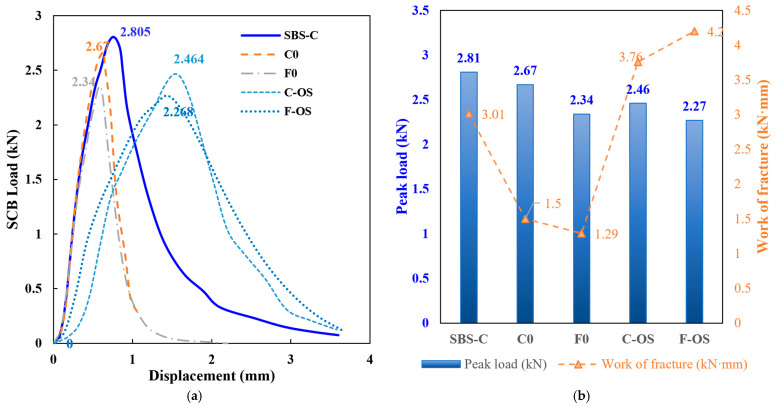
(**a**) SCB load–displacement curves and (**b**) SCB fracture parameters of selected WPA–SBS asphalt mixtures.

Figure 11 presents the SCB load–displacement curves of the selected WPA–SBS asphalt mixtures. The curves show clear differences in peak load, post-peak softening, and residual fracture resistance. The SBS-C mixture reached a relatively high peak load of 2.81 kN at a displacement of 0.76 mm, followed by a sharp post-peak reduction. This indicates that the control SBS mixture had good crack-initiation resistance, but its fracture process was relatively abrupt after the peak load.

The untreated WPA mixtures showed more brittle fracture behavior. C0 reached a peak load of 2.67 kN at 0.64 mm, then dropped rapidly to 1.38 kN at 0.79 mm and 0.84 kN at 0.90 mm. This sharp reduction indicates limited post-peak resistance. F0 showed a slightly different response, with a peak load of 2.34 kN at 0.60 mm, followed by a very abrupt drop to 1.71 kN at 0.70 mm and 0.99 kN at 0.82 mm. The rapid loss of load-carrying capacity suggests that untreated WPA weakened crack-propagation resistance, especially for the fine WPA system.

The C-OS mixture showed a lower early stiffness but a broader and more stable fracture response. Its peak load reached 2.46 kN at 1.52 mm, which occurred at a much larger displacement than SBS-C and C0. Although its peak load was slightly lower than the control and C0, the delayed peak and longer post-peak tail indicate higher deformation tolerance and better fracture energy absorption. This behavior suggests that AS-OS improved the WPA–binder interface and reduced sudden crack propagation.

F-OS also showed improved ductility compared with F0. The peak load reached 2.27 kN at 1.44 mm, and the load decreased more gradually after the peak. Compared with F0, the peak displacement increased from 0.60 mm to 1.44 mm, indicating a much more ductile cracking response. Although F-OS did not have the highest peak load, its broader curve and longer softening branch indicate better resistance to crack growth after initiation.

These results show that peak load alone is not sufficient to evaluate cracking resistance. SBS-C and C0 had relatively high peak loads, but their post-peak drops were sharp. In contrast, C-OS and F-OS showed lower or comparable peak loads with much larger displacement at peak and more gradual post-peak softening. This trend agrees with the adhesion and shear results, where AS-OS improved interfacial stability. The improved fracture behavior is likely related to better binder retention around WPA particles, allowing the mixture to absorb more energy during crack propagation.

The SCB results are consistent with related studies on polymer-modified asphalt mixtures, where improved interfacial bonding and more stable binder films often increase fracture tolerance even when peak strength is not maximized. In this study, AS-OS shifted the fracture response from a brittle, strength-controlled behavior to a more ductile, energy-absorbing behavior. Therefore, the SCB results further support AS-OS as the most effective anti-stripping agent for improving the cracking resistance of WPA-containing SBS-modified asphalt mixtures.

The fracture energy results further confirm that peak load alone did not fully represent cracking resistance. Although SBS-C showed the highest peak load, its work of fracture was 3.01 kN·mm because of the abrupt post-peak drop. In contrast, C-OS and F-OS showed higher fracture work values of 3.76 and 4.20 kN·mm, corresponding to increases of 24.8% and 39.5% relative to SBS-C. This indicates that AS-OS improved post-cracking energy absorption and delayed crack propagation in the WPA mixtures. The untreated C0 and F0 mixtures showed much lower fracture work, confirming their brittle response and weaker WPA–binder interface.

### 3.8. Normalized Evaluation of WPA and Anti-Stripping Effects

To better distinguish the effect of WPA itself from the effect of anti-stripping treatment, the main performance indicators were normalized to a 0–1 scale, as summarized in Table 8. For properties where a higher value indicates better performance, including BBS, tensile bond strength, shear strength, shear energy, TSR, and SCB fracture work, the highest value was assigned as 1.0 and the lowest value as 0. For properties where a lower value indicates better performance, including HWT rut depth and MSCR accumulated strain, the normalization was reversed. Therefore, a normalized value close to 1.0 indicates better performance, while a value close to 0 indicates weaker performance.

The normalized results show that the untreated WPA mixtures had the lowest overall performance indices. C0 and F0 had average indices of 0.12 and 0.05, respectively, confirming that WPA itself reduced adhesion, moisture resistance, deformation resistance, and fracture performance when no anti-stripping agent was used. The lower index of F0 also confirms that fine WPA was more sensitive to interfacial weakness and moisture-related damage. After AS-OS treatment, the average indices increased sharply to 0.95 for C-OS and 0.91 for F-OS. This indicates that AS-OS effectively compensated for the negative effect of WPA by improving WPA–SBS interfacial compatibility. Therefore, the performance of WPA mixtures depends not only on the use of plastic aggregate itself, but also on whether the WPA–binder interface is properly treated.

### 3.9. Limitations and Future Study

This study evaluated LDPE-rich WPA containing a PE/PP mixed plastic fraction and inorganic additive/filler. Other plastic aggregates, such as PET-, HDPE-, PP-, PVC-, or rubber-based aggregates, may have different surface polarity, stiffness, melting behavior, and binder affinity. Therefore, the applicability of AS-OS and other anti-stripping agents to different plastic aggregate types should be verified separately in future work.

Although AS-OS showed the best overall performance, its interfacial mechanism was inferred from mechanical evidence rather than directly measured. The improvements in BBS, tensile bond strength, shear bond strength, TSR, HWT, MSCR, and SCB results indicate better WPA–SBS interface performance, but they do not directly confirm chemical interaction, surface energy change, or binder coating morphology. Therefore, the next stage of this research should include direct interface characterization, such as contact angle and surface free energy analysis to evaluate wetting and adhesion work, FTIR spectroscopy to identify possible functional group interactions after AS-OS treatment, and SEM or other microscopic observations to examine binder coating continuity, interfacial morphology, and stripping damage after moisture conditioning. These additional tests would provide stronger mechanistic evidence for the role of organosilane-based anti-stripping agents in improving WPA–SBS asphalt mixture performance.

## 4. Conclusions

This study evaluated the compatibility of three liquid anti-stripping agents with coarse and fine WPA in SBS-modified asphalt mixtures. The main conclusions are as follows:

Untreated WPA reduced the initial adhesion capacity of the SBS binder. The BBS decreased from 552 kPa for NA-SBS to 318 kPa for C0 and 286 kPa for F0, corresponding to reductions of 42.4% and 48.2%, respectively. This confirms that the polymeric WPA surface weakened binder adhesion, especially for the fine WPA system.

- Among the three anti-stripping agents, AS-OS showed the strongest interface improvement. It increased BBS by 52.8% for WPA-C and 61.5% for WPA-F. At the optimum dosage of 0.5%, AS-OS also increased tensile bond strength to 0.76 MPa for C-OS and 0.71 MPa for F-OS, corresponding to improvements of 81.0% and 97.2% over the untreated systems.
- AS-OS also improved shear resistance and interface durability. The peak shear strength increased from 0.68 to 1.08 MPa for WPA-C and from 0.60 to 1.01 MPa for WPA-F. The shear energy increased by 108.5% for C-OS and 132.4% for F-OS. The failure mode also shifted from adhesive failure to mixed or cohesive/mixed failure, indicating improved WPA–SBS interfacial compatibility.
- The mixture-scale tests confirmed that the improved interface response was transferred to better pavement performance. AS-OS increased TSR from 77.4% to 89.0% for WPA-C and from 73.4% to 86.2% for WPA-F. In the HWT test, C-OS and F-OS reduced the final rut depth by 44.0% and 45.8%, respectively, compared with their untreated WPA mixtures.
- The MSCR and SCB results further confirmed the benefit of AS-OS treatment. At 3.2 kPa, C-OS and F-OS reduced the final MSCR strain by 35.8% and 35.1%, respectively. In the SCB test, the work of fracture increased from 3.01 kN·mm for SBS-C to 3.76 kN·mm for C-OS and 4.20 kN·mm for F-OS, indicating improved crack-propagation resistance.

Overall, AS-OS was the most effective anti-stripping agent for the LDPE-rich WPA–SBS asphalt mixtures evaluated in this study. It improved adhesion, shear resistance, moisture resistance, rutting resistance, creep–recovery behavior, and fracture performance. The results show that WPA use in SBS-modified asphalt mixtures should be supported by compatibility-based anti-stripping agent selection, rather than relying only on WPA replacement content.

## Figures and Tables

**Table 1:** Basic properties of SBS-modified asphalt binder.

Property	Unit	Test Method	Value
Penetration at 25 °C	0.1 mm	ASTM D5 [39]	52
Softening point	°C	ASTM D36 [40]	76.5
Ductility at 25 °C	cm	ASTM D113 [41]	>100
Specific gravity at 25 °C	—	ASTM D70 [42]	1.035
Rotational viscosity at 135 °C	Pa·s	ASTM D4402 [43]	1.82
Rotational viscosity at 165 °C	Pa·s	ASTM D4402 [43]	0.54
Elastic recovery at 25 °C	%	ASTM D6084 [44]	87
PG grade	—	AASHTO M 320 [45]	PG 76-22

**Table 2:** Physical properties of natural aggregates and mineral filler.

Material	Bulk Specific Gravity	SSD Specific Gravity	Apparent Specific Gravity	Water Absorption (%)	Los Angeles Abrasion (%)
Coarse aggregate 20 mm	2.721	2.734	2.757	0.48	25.85
Coarse aggregate 13 mm	2.681	2.708	2.756	1.02	32.97
Fine aggregate	2.694	2.749	2.851	2.04	—
Limestone filler	—	2.765	—	—	—

**Table 3:** Physical properties of WPA used in this study.

Property	Unit	WPA-C	WPA-F
Main particle size	mm	5–13	<5
Bulk density	g/cm^3^	0.50	0.50
Specific gravity	—	1.36	1.36
Solid content	%	36.8	36.8
Water absorption	%	0.18	0.24
Fineness modulus	—	6.41	4.16

**Table 4:** Chemical and polymeric properties of waste plastic aggregates used in this study.

Property	Unit	WPA-C	WPA-F
Main recycled polymer type	—	LDPE-rich recycled plastic	LDPE-rich recycled plastic
LDPE content	wt.%	72.5	70.8
PP content	wt.%	11.2	12.6
PE/PP mixed plastic fraction	wt.%	83.7	83.4
Inorganic additive/filler	wt.%	12.8	13.5
Residual impurities	wt.%	3.5	3.1
Ash content	wt.%	13.1	13.8
Melting range	°C	108–132	108–134
Softening tendency during asphalt mixing	—	Stable at 160 °C	Stable at 160 °C

**Table 5:** Chemical and physical properties of liquid anti-stripping agents used in this study.

Agent Code	Chemical Class	Main Chemical/Functional Basis	Specific Gravity at 25 °C	Viscosity at 25 °C (mPa·s)	Flash Point (°C)	Chemical Index	Active Content (%)
AS-Am	Amine-based liquid anti-stripping agent	Fatty amidoamine derivative with polar amine group and hydrophobic hydrocarbon chain	0.92	320	145	Amine value: 245 mg KOH/g	58
AS-OS	Organosilane coupling-type adhesion promoter	Organofunctional alkoxysilane blend with hydrolysable silane group and organic functional segment	0.98	42	105	pH after hydrolysis: 5.6	46
AS-Es	Ester/surfactant-based wetting agent	Fatty acid ester and nonionic surfactant blend with surface-active ester segment	0.94	185	168	Acid value: 4.8 mg KOH/g; HLB: 11.2	72

**Table 6:** Mixture design and component proportions of selected WPA–SBS asphalt mixtures.

Mix Code	SBS Binder (%)	Coarse NA (%)	Fine NA (%)	Mineral Filler (%)	WPA-C (%)	WPA-F (%)	Anti-Stripping Agent	Agent Dosage (% by Binder wt.)
SBS-C	5.4	52.0	40.0	2.6	0.0	0.0	None	0.0
C0	5.4	46.8	40.0	2.6	5.2	0.0	None	0.0
F0	5.4	52.0	36.0	2.6	0.0	4.0	None	0.0
C-Am	5.4	46.8	40.0	2.6	5.2	0.0	AS-Am	0.5
C-OS	5.4	46.8	40.0	2.6	5.2	0.0	AS-OS	0.5
C-Es	5.4	46.8	40.0	2.6	5.2	0.0	AS-Es	0.5
F-Am	5.4	52.0	36.0	2.6	0.0	4.0	AS-Am	0.5
F-OS	5.4	52.0	36.0	2.6	0.0	4.0	AS-OS	0.5
F-Es	5.4	52.0	36.0	2.6	0.0	4.0	AS-Es	0.5

**Table 7:** Mixing and compaction conditions for WPA–SBS asphalt mixtures.

Process	Parameter	Condition
Aggregate drying	Temperature/time	105 ± 5 °C/24 h
Natural aggregate preheating	Temperature	170 ± 5 °C
WPA preheating	Temperature	120 ± 5 °C
SBS binder heating	Temperature	160 ± 5 °C
Anti-stripping blending	Dosage	0.3%, 0.5%, 0.7% by binder weight
Anti-stripping blending	Temperature/speed/time	155 ± 5 °C/500 rpm/10 min
Asphalt mixing	Temperature	160 ± 5 °C
Asphalt mixing	Speed/time	120 rpm/3 min
Short-term conditioning	Temperature/time	135 ± 5 °C/2 h
Compaction	Temperature	145 ± 5 °C
Marshall specimens	Compaction effort	75 blows per side
HWT and SCB specimens	Target air voids	7.0 ± 1.0%

**Table 8:** Normalized performance index of untreated and treated WPA–SBS asphalt mixtures.

Mix Code	BBS	Tensile Bond	Shear Strength	Shear Energy	TSR	HWT Rutting	MSCR Strain	SCB Fracture Work	Average Index
C0	0.16	0.15	0.17	0.14	0.26	0	0	0.07	0.12
F0	0	0	0	0	0	0.24	0.19	0	0.05
C-OS	1	1	1	1	1	0.84	0.9	0.85	0.95
F-OS	0.88	0.87	0.85	0.87	0.82	1	1	1	0.91
F-Es	0.71	0.73	0.71	0.75	0.72	0.83	0.75	—	0.74

Note: HWT rutting and MSCR strain were normalized as lower-is-better indicators. The average index was calculated using the available normalized indicators. The SCB fracture work of F-Es was not included because this mixture was not evaluated in the SCB dataset.

## Data Availability

The original contributions presented in this study are included in the article. Further inquiries can be directed to the corresponding author.
